# Mechanistic Modulation of Lipopolysaccharide-Induced Hepatic Injury by Chitosan-Coated Selenium Nanoparticles: Targeting the STEAP-3/TLR-4 and IL-17/TRAF-6/HSP-90 Axes

**DOI:** 10.3390/pharmaceutics18030388

**Published:** 2026-03-20

**Authors:** Asmaa Ramadan, Eman Hamza, Eman Ali Elkordy, Eslam E. Abd El Fattah, Amr Yehia, Ahmed S.G. Srag El-Din

**Affiliations:** 1 Department of Biochemistry, Faculty of Pharmacy, Delta University for Science and Technology, Gamasa 11152, Egypt; asmaa.esmail@deltauniv.edu.eg (A.R.); or eslam_620@yahoo.com (E.E.A.E.F.); 2 Department of Biochemistry, College of Medicine, Imam Mohammad Ibn Saud Islamic University (IMSIU), Riyadh 13317, Saudi Arabia; 3 Department of Anatomy & Physiology, College of Medicine, Imam Mohammad Ibn Saud Islamic University (IMSIU), Riyadh 13317, Saudi Arabia; eaelkordy@imamu.edu.sa; 4 Beckman Research Institute, City of Hope, Duarte, CA 91010, USA; 5 Morsani College of Medicine, University of South Florida, 12901 Bruce B. Downs Blvd., Tampa, FL 33612, USA; amr126@usf.edu; 6 Department of Pharmaceutics, Faculty of Pharmacy, Delta University for Science and Technology, Gamasa 11152, Egypt

**Keywords:** selenium nanoparticles, chitosan, acute liver injury, lipopolysaccharide, TLR-4

## Abstract

**Background/Objectives**: The aim of the current study was to investigate the mechanistic hepatoprotective efficacy of selenium (SE) and chitosan-coated selenium nanoparticles (CS-SENPs) using a rat model induced by lipopolysaccharide (LPS). **Methods**: CS-SENP was prepared and characterized for particle size, polydispersity index (PDI), zeta potential, transmission electron microscope (TEM), and Fourier transform infrared spectroscopy (FTIR). Male albino rats (*n* = 40) were divided into four groups: control, LPS, SE, and CS-SENP. SE and CS-SENPs (5 mg/kg orally for 14 days) were given before LPS injection. Tissue architecture was assessed using histopathological analysis. HSP-47 and STEAP-3 protein expression levels were measured using ELISA, and oxidative stress markers were quantitatively evaluated. The expression of HO-1, TLR-4, STAT-3, TRAF-6, and IL-17A was measured using immunohistochemical analysis. Furthermore, HSP-90 expression was evaluated by immunofluorescence labeling. **Results**: CS-SENP characterization revealed uniform (PDI = 0.125 ± 0.04) nanoparticle size (108.54 ± 2.24 nm), with high zeta potential (+63.92 ± 6.287 mV), attributed to the CS layer, which was confirmed by FTIR and TEM as an electron-lucent halo enveloping the individual SENP cores. CS-SENPs significantly reduced lipid peroxidation (MDA) and restored glutathione (GSH) more effectively than SE. CS-SENPs improved redox (upregulated HO-1) and iron balance (downregulated STEAP-3), and also increased the anti-inflammatory effect (suppressed TLR-4, IL-17A, TRAF-6, and STAT-3). CS-SENPs showed superior antifibrotic efficacy (suppresses stress proteins, HSP-47 and HSP-90). Rats treated with CS-SENPs had nearly normal liver structure. **Conclusions**: The results concluded that CS-SENPs had superior and multi-targeted hepatoprotection against LPS-induced liver damage.

## 1. Introduction

Acute liver injury (ALI) is a rapid-onset syndrome caused by diverse insults, including autoimmune reactions, ischemia–reperfusion injury, drug-induced liver injury, and viral infections. Despite varied etiologies, ALI shares a common pathogenesis marked by intense inflammation, accelerated hepatocyte death, and disruption of hepatic architecture [1,2]. ALI’s high morbidity and complex signaling highlight the need to define the mechanisms coordinating injury and regeneration. Such mechanistic insight is essential for developing targeted therapies that limit hepatocellular damage and support endogenous repair. Accordingly, examining key immunomodulatory mediators is vital for identifying the molecular drivers of hepatic inflammation and its potential progression toward fibrosis.

A crucial cytokine in the control of the hepatic immune system in several liver disorders is interleukin-17 (IL-17). By encouraging the release of inflammatory cytokines and chemokines, IL-17, which is secreted by T helper 17 (Th17) cells and innate lymphoid cells, contributes to liver damage. Increased IL-17 signaling increases hepatic inflammation and promotes immune cell infiltration into the liver parenchyma, particularly in metabolic dysfunction-associated steatotic liver disease [3]. IL-17 directly stimulates fibrogenesis in addition to systemic inflammation. Mechanistically, IL-17 increases hepatocyte mortality and inflammation by intensifying immune-mediated liver injury via the signal transducer and activator of transcription-3 (STAT-3)/IFI16 axis [3]. Targeting the IL-17 signaling axis or its upstream transcriptional regulators, such as retinoic acid-related orphan receptor gamma t, has shown remarkable therapeutic potential in preclinical studies. In experimental models of liver illness, altering these targets has been demonstrated to effectively lower immune cell infiltration and lessen hepatic harm [4,5]. Collectively, these striking findings demonstrate IL-17’s well-established dual pathogenic role as a key mediator of both inflammation and fibrogenesis and show it to be a viable and clinically significant therapeutic target for a variety of liver diseases [6].

A crucial adaptor in IL-17 signaling during hepatic damage is tumor necrosis factor receptor-associated factor-6 (TRAF-6). The adaptor protein Act1 (CIKS) is drawn in when IL-17 binds to its receptor complex (IL-17RA/RC), which subsequently promotes TRAF-6 activation. Initiating downstream proinflammatory pathways that intensify hepatic inflammation depends on this signaling event [7]. Six-transmembrane epithelial antigen of prostate-3 (STEAP-3) has become an important enzymatic mediator that goes beyond traditional cytokine signaling. Ferroptosis, a type of iron-dependent controlled necrotic cell death caused by lipid peroxidation, is inextricably tied to STEAP-3, an important ferrireductase that controls cellular iron metabolism. It is becoming more widely acknowledged that STEAP-3-regulated ferroptosis plays a crucial role in causing severe liver damage and the next stages of hepatic tissue healing [8]. Furthermore, accumulating evidence points to STEAP-3 as a therapeutic target in liver damage and fibrosis related to hepatitis.

An important micronutrient for maintaining redox balance, selenium (SE) is a cofactor in selenoproteins such as thioredoxin reductase (TrxR) and glutathione peroxidase (GPx) [9,10]. Higher SE status has been associated with a lower incidence of advanced liver diseases such as cirrhosis and hepatocellular carcinoma [11,12]. Liang, Huang [13] showed that dietary SE intake and circulating SE concentration affect liver function parameters. On the other hand, Wang, Seo’s [14] results show that there are non-linear relationships between serum SE levels and both the prevalence of NAFLD and ALT activity, with positive correlations only observed above a serum SE level of 130 μg/L. Despite the physiological importance of SE, conventional SE species, such as selenites and organic SE, have a narrow therapeutic window, poor bioavailability in oral formulations, and rapid systemic clearance, limiting its clinical application at hepatoprotective doses [15,16].

To circumvent these pharmacological limitations, Selenium Nanoparticles (SENPs) have emerged as a prominent candidate in nanomedicine. Due to their unique nanometric dimensions, SENPs demonstrate greater bioavailability and potent antioxidant properties with significantly reduced toxicity compared to traditional SE forms [17]. The smaller particle size promotes slower SE release and reduces the systemic toxicity associated with high-dose bulk selenium, while maintaining potent antioxidant activity through GPx and TrxR activation [18,19]. Compared to inorganic and organic selenocompounds, SENPs demonstrate better biocompatibility, increased bio-efficacy, and decreased toxicity [14,15], making them a superior selenium delivery platform for hepatic applications [20,21].

However, the clinical translation of bare SENPs is often hindered by their high surface energy, leading to rapid aggregation and chemical instability (oxidation) in complex biological media. To address these stability challenges, the strategic functionalization of SENPs with Chitosan (CS) has been widely adopted. CS, a naturally cationic polysaccharide, was selected as a coating material for SENPs based on its distinct advantages for hepatic drug delivery. The protonated amino groups of CS allow strong electrostatic interactions with negatively charged cell membranes and serum components, facilitating uptake by liver cells and the reticuloendothelial system [22,23,24]. Kupffer cells and other hepatic phagocytes recognize CS’s positive surface charge, causing preferential accumulation in hepatic tissue and passively elevating local drug levels, a form of passive liver targeting particularly beneficial for hepatocellular carcinoma, fibrosis, and inflammatory liver disorders [25,26]. Furthermore, CS provides colloidal stability to SENPs, preventing aggregation and extending circulatory half-life [27].

The present study selected the following signaling pathways based on their established mechanistic roles in LPS-induced hepatic injury: (1) TLR-4 is the primary pattern recognition receptor for LPS, serving as the initiating trigger of the inflammatory cascade via NF-κB and MAPK activation; (2) IL-17/TRAF-6 constitutes a downstream adaptive immune amplification axis, promoting neutrophil recruitment, hepatic inflammation, and fibrogenesis; (3) STAT-3 represents a convergence node for cytokine-driven transcription, supporting hepatocellular survival, sustained inflammation, and fibrosis in the context of IL-17 signaling; (4) STEAP-3 is a ferrireductase that links iron dysregulation to ferroptosis—a form of iron-dependent, lipid peroxidation-driven cell death increasingly recognized as a key mechanism in acute hepatic injury; and (5) HSP-47 and HSP-90 are molecular chaperones that mark the stress-protein and fibrogenic response networks activated during endotoxemia. These pathways are mechanistically interconnected and collectively drive LPS-induced liver damage at multiple molecular levels, providing a comprehensive framework for evaluating multi-targeted hepatoprotection of chitosan-coated selenium nanoparticles (CS-SENPs).

The current study’s hypothesis was to functionalize SENPs with CS to increase hepatoprotective efficacy. This strategy looks to reduce ALI by using the complementary qualities of CS’s advantageous biodistribution and mediated uptake and SENPs’ antioxidant capacity. While prior studies have examined selenium nanoparticles or chitosan-coated carriers in general inflammatory or hepatotoxicity models, the present study is, to our knowledge, the first to: (1) simultaneously interrogate the STEAP-3/TLR-4 and IL-17/TRAF-6/HSP-90 axes in LPS-induced hepatic injury within a single experimental model; (2) link CS-SENP treatment to ferroptosis regulation via STEAP-3 modulation in the liver; and (3) employ a multimodal quantitative approach combining ELISA, immunohistochemistry, and immunofluorescence to map mechanistic hepatoprotection across redox, iron metabolism, inflammatory, and chaperone pathways simultaneously. The study intends to find new therapeutic targets and pathways that regulate the equilibrium between liver injury and regeneration by combining these mechanistic insights.

## 2. Materials and Methods

### 2.1. Materials

Sodium selenite was acquired from LANXESS AG (Kennedy Platz 1, Cologne, Germany); chitosan (MWT 100,000–300,000) was purchased from Across Organics, Fair Lawn, NJ, USA; ascorbic acid, analytical grade; and LPS (from *Escherichia coli* O55:B5) was acquired from Sigma-Aldrich in St. Louis, MO, USA.

### 2.2. Chitosan-Coated Selenium Nanoparticle Preparation

CS-SENPs were prepared following the procedure described by Srag El-Din, Yehia et al. [28]. Ascorbic acid was used to chemically reduce sodium selenite while CS served as a stabilizing agent. Three stock solutions were made prior to the synthesis: 100 mM ascorbic acid, 50 mM sodium selenite, and 1% (*w*/*w*) CS, which was made by dissolving 50 mg of CS in 5 mL of 4% (*v*/*v*) acetic acid.

In amber-colored glassware to prevent oxidation, 2 mL of the sodium selenite was vigorously swirled with 1 mL of 1% (*w*/*w*) CS solution, then 1 mL of 100 mM ascorbic acid solution was added dropwise. After that, the 4 mL reaction mixture was diluted to a total volume of 10 mL with deionized water, and the system was stirred for an hour at room temperature. The resulting reddish-orange suspension was used directly without further purification. To ensure the reproducibility of the formulation, three independent batches of CS-SENPs were prepared and characterized, with data expressed as the mean ± standard deviation (SD).

### 2.3. Particle Size, Polydispersity Index Determination, and Zeta Potential

A Malvern size distribution analyzer (Malvern Instruments, Malvern, Worcestershire, UK) was used to measure the particle size (PS), polydispersity index (PDI), and zeta potential of CS-SENPs in aqueous solution at 25 °C. To maintain colloidal stability and ensure repeatability, all experiments were conducted in triplicate using deionized water as the dispersant. Mean ± standard deviation (SD) is used to express the results [29].

### 2.4. Transmission Electron Microscopy

The morphology of the CS-SENPs was examined using transmission electron microscopy (TEM) (JEM-2100, JEOL, Tokyo, Japan). A droplet of the CS-SENP suspension was stained with 1% phosphotungstic acid on a dry carbon-coated copper grid before being let to air dry. The built grid was then seen and imaged using TEM [30].

### 2.5. Fourier-Transform Infrared Spectroscopy

After being lyophilized with a vacuum freeze-drier (SIM International Group, Charlotte, NC, USA), the CS-SENP samples were examined using Fourier transform infrared (FTIR) spectroscopy (BRUKER, IFS 66, Karlsruhe, Germany). For FTIR examination, 1–2 mg of each sample was finely ground, blended with potassium bromide, and pressed into disks for spectral analysis. The FTIR spectra were recorded over a scanning range of 4000 to 400 cm^−1^ [31].

### 2.6. Experimental Design

Forty male albino rats weighing between 120 and 140 g were acquired from the National Research Center in Doki, Giza, Egypt. Rats were randomly assigned to groups using a simple randomization table generated with a random number generator. Animals were maintained in standard polypropylene cages (5 per cage) under controlled temperature (25 ± 2 °C), relative humidity (55 ± 10%), and a 12 h light/dark cycle with ad libitum access to standard rodent pellets and water. Bedding was changed twice weekly.

All animal experiments were designed and conducted in accordance with the Declaration of Helsinki, and all procedures were approved by the Institutional Animal Care and Use Committee of the Faculty of Pharmacy, Delta University for Science and Technology (approval code: FPDU32/2025). The approval for code FPDU32/2025 was granted on 30 November 2025. Four groups of ten rats each were established from the rats: Rats in Group 1 (normal control, NC) were given normal saline (2 mL/kg P.O.) every day for two weeks. Rats in Group 2 (LPS) were given LPS intraperitoneally once at a dose of 5 mg/kg. Rats in group 3 (SE group) were given SE (5 mg/kg P.O.) every day for two weeks. On the fourteenth day, they were given LPS, the same as in group 2. Rats in Group 4 (CS-SENP group) were given CS-SENPs (5 mg/kg P.O.) every day for two weeks. On the fourteenth day, they were given LPS, the same as in Group 2. The oral dose of 5 mg/kg for both SE and CS-SENPs was selected based on previous published studies [32,33,34,35]. At the end of the experiment, all rats were anesthetized with phenobarbital (50 mg/kg, intraperitoneally), euthanized by cervical dislocation 24 h after LPS administration, and their livers were dissected.

### 2.7. Collection of Blood Samples and Liver Tissues

The livers were dissected, and blood samples were collected simultaneously. Following blood collection, plasma was extracted and kept for biochemical analysis at −80 °C. The just-removed livers were cleaned with a fresh paper towel and rinsed with ice-cold saline. The liver was divided into two sections: one was retained in a 10% neutral buffered formalin solution for immunofluorescence and histological analysis, and the other was frozen in liquid nitrogen right away and stored at −80 °C for the enzyme-linked immunosorbent test (ELISA).

### 2.8. Histopathological Examination

In the Pathology Department of the Faculty of Veterinary Medicine at Mansoura University in Egypt, liver tissues were preserved in formalin, embedded in paraffin, sliced into five-micrometer-thick slices, and stained with hematoxylin and eosin (H & E). A pathologist used a light microscope to view the slides while being blindfolded, and a computerized image-capture device was used to collect the images.

### 2.9. Biochemical Analysis

#### 2.9.1. Assessment of Oxidative Stress Markers

Lipid peroxidation in liver tissue was assessed using the Malondialdehyde (MDA) colorimetric assay kit (Biodiagnostics, Giza, Egypt) and the thiobarbituric acid assay adapted in accordance with the method of Draper and Hadley [36]. Additionally, a commercial kit (Biodiagnostics, Egypt) was used to assess the absorbance at 405 nm to estimate liver glutathione (GSH) using a colorimetric assay method.

#### 2.9.2. ELISA Assessment for HSP-47 and STEAP-3

HSP-47 and STEAP-3 concentrations in liver tissue were measured using rat HSP-47 ELISA kits (Finetest Co., Ltd., Wuhan, China) and STEAP-3 (INNOVA BIOTECH Co., Ltd., Beijing, China) in accordance with the manufacturer’s protocol and expressed as ng/mg tissue.

#### 2.9.3. Immunohistochemical Detection of HO-1, TLR-4, STAT-3, TRAF-6, and IL-17A

Immunohistochemical analysis of heme oxygenase-1 (HO-1), TLR-4, STAT-3, TRAF-6, and IL-17A was performed using paraffin-embedded liver sections. The serial tissue sections were dewaxed and rehydrated, followed by antigen retrieval and then treated with 0.3% hydrogen peroxide for 15 min and subsequently incubated in a protein-blocking solution for 30 min at room temperature. Sections were then incubated overnight with the respective primary antibodies (HO-1, TLR-4, STAT-3, TRAF-6, and IL-17A). After washing three times with phosphate-buffered saline, the slides were treated with the proper secondary antibodies for 30 min at room temperature (anti-mouse or anti-rabbit IgG). Immunoreactivity was visualized using a commercial diaminobenzidine (DAB) detection kit (Liquid DAB + Substrate Chromogen System; Dako, Nowy Sącz, Poland), followed by counterstaining with Mayer’s hematoxylin. Normal mouse serum was used in place of the primary antibody as a negative control.

Cells exhibiting cytoplasmic staining for IL-17A and/or TRAF-6 were considered positive. Semi-quantitative evaluation of positive staining was conducted according to the modified Allred scoring system. The percentage of positive area across the entire liver section was assessed under low power (10×) and assigned a score from 0 to 5 (1 = <10%; 2 = 10–20%; 3 = 20–50%; 4 = 50–70%; 5 = >70%). Staining intensity was graded as 1 = weak, 2 = moderate, or 3 = strong. The two scores were summed to obtain a final immunohistochemical grade. For HO-1, TLR-4, and STAT-3, the labeling index was expressed as the percentage of positive cells per total counted cells in seven to nine high-power fields.

#### 2.9.4. Immunofluorescence Staining of HSP-90

Immunofluorescence staining of formalin-fixed, paraffin-embedded rat liver tissue sections were made using HSP-90 primary antibody (HSP-90 Monoclonal Antibody; clone H9010, FITC). A four-tier approach was used to grade the strength of the IF staining: 0 for no staining, 1+ for weak, 2+ for moderate, and 3+ for strong. To put it briefly, each sample’s H-score was determined by multiplying the percentage of positive cells (0–100%) by the total of each intensity (0–3). The range of the score was 0 to 300. The H-score’s median value was determined [37].

### 2.10. Statistical Analysis

GraphPad Prism software version 6 (GraphPad Software Inc., La Jolla, CA, USA) was used to statistically analyze the data, and the results are presented as the mean ± SD. Groups were compared using a one-way analysis of variance (ANOVA) and then a Tukey–Kramer post hoc multiple comparisons test to identify statistically significant pairwise differences between all group combinations. Statistical significance was defined as a *p* value < 0.05.

## 3. Results

### 3.1. Chitosan-Coated Selenium Nanoparticle Preparation and Characterization

**Figure 1:**
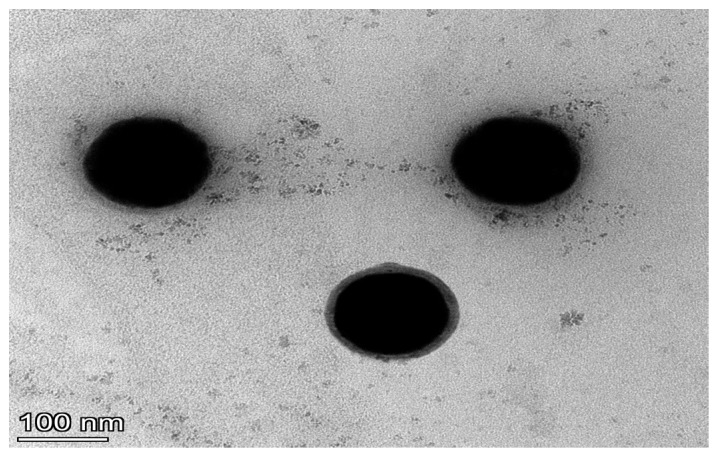
TEM of CS-SENPs. TEM showing an electron-lucent CS corona surrounding the dense SENP cores, indicative of effective surface functionalization and colloidal stabilization.

**Figure 2:**
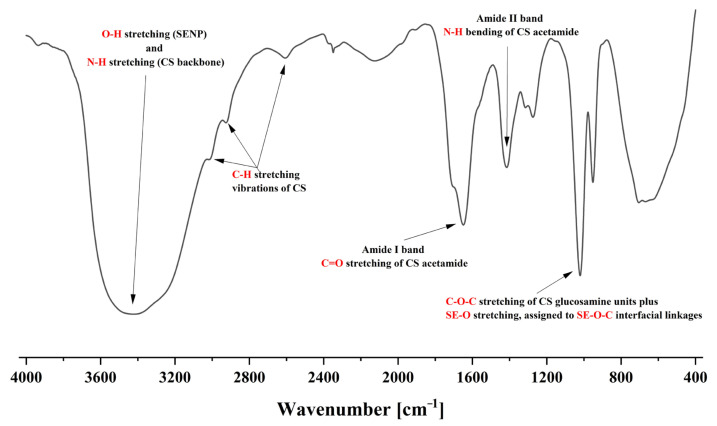
FTIR spectrum of CS-SENPs. The FTIR spectrum highlighting a broad band at 3426 cm^−1^ attributed to overlapping O-H and N-H stretching vibrations of the CS backbone, alongside C-H stretching bands at 3024, 2926, and 2607 cm^−1^ that further confirm the presence of CS on the nanoparticle surface. Distinct amide I and amide II bands at 1648.84 and 1415.49 cm^−1^, respectively, indicate preserved acetamide functionalities within the CS coating, while an intense band around 1100 cm^−1^, arising from C-O-C and SE-O stretching, suggests partial selenium surface oxidation and/or formation of SE–O–C interfacial linkages consistent with strong CS-SENP interactions.

The PS of CS-SENPs was 108.54 ± 2.24 nm with a PDI of 0.125 ± 0.04, suggesting a highly monodisperse nanoparticle distribution. The observed zeta potential was a strong positive value of 63.92 ± 6.287 mV, indicating exceptional colloidal stability due to high electrostatic repulsion. The DLS results were further supported by TEM imaging (Figure 1), which showed uniformly distributed, spherical nanoparticles with smooth surfaces. The SENP core was surrounded by a characteristic CS layer that formed a polymeric corona and was evident as an electron-lucent halo. This polymeric corona is consistent with effective surface functionalization and illustrates the crucial role of CS as a stabilizing agent in preventing nanoparticle aggregation. The overlapping stretching vibration of the O-H group from SENPs and the N-H group found in the CS polysaccharide backbone was responsible for the clear broad absorption band at 3426 cm^−1^ in the FTIR spectra of CS-SENPs (Figure 2). The presence of CS macromolecular chains on the SENP core was indicated by the detection of distinctive C-H stretching vibrations at 3024, 2926, and 2607 cm^−1^. The structural integrity of the CS’s acetamide functionalities inside the coating matrix is demonstrated by the absorption bands at 1648.84 cm^−1^ and 1415.49 cm^−1^, which correspond to amide I and amide II vibrations and reflect C=O stretching and N-H bending vibrations, respectively. A prominent absorption band at approximately 1100 cm^−1^ was detected, arising from overlapping C-O-C stretching vibrations of the glucosamine units in CS and SE-O stretching modes, signifying the formation of covalent SE-O-C interfacial linkages and confirming successful interaction between CS and the SENP surface.

### 3.2. Effect of SE and CS-SENP Treatment on Histopathological Changes in the Rat’s Liver

**Figure 3:**
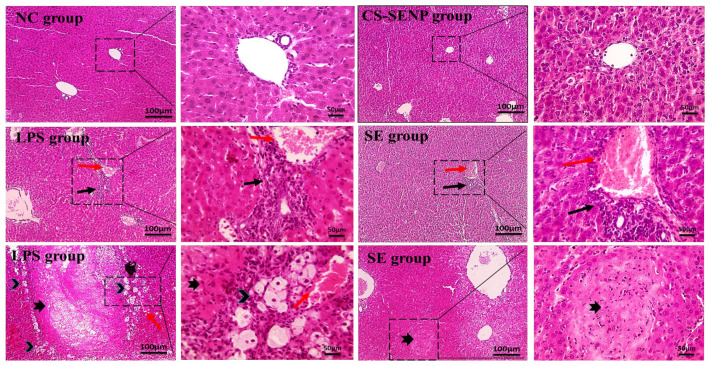
Liver sections showing normal hepatic cords, central veins, portal areas, and sinusoids in the control group and the groups that received SE. Liver sections from the LPS group showing portal inflammation & fibrosis (thin black arrow) and congestion (red arrows). Moreover, a large area of necrosis (thick black arrow) is seen in hepatic parenchyma surrounded by ballooning degeneration of adjacent hepatocytes (black arrowheads) and congested blood vessels (red arrows). Liver sections from the SE group showing mild portal inflammation (thin black arrow) and congestion (red arrows). A small focal area of necrosis (thick black arrow) is seen in hepatic parenchyma. H&E low magnification ×: 100 bar 100, high magnification ×: 400 bar 50.

Histopathological examination of hepatic tissue (Figure 3) revealed that control and CS-SENP-treated livers exhibited normal architecture with preserved parenchymal integrity. In contrast, livers from the LPS-treated group demonstrated marked pathological alterations, including hepatic congestion, portal inflammation, fibrosis, and widespread necrosis surrounded by clogged vessels and hepatocyte ballooning. The SE-treated group showed a moderate reduction in portal inflammation and only focal, small necrosis.

### 3.3. Effect of SE and CS-SENP Treatment on MDA, GSH, HSP-47, and STEAP-3 Levels

**Figure 4:**
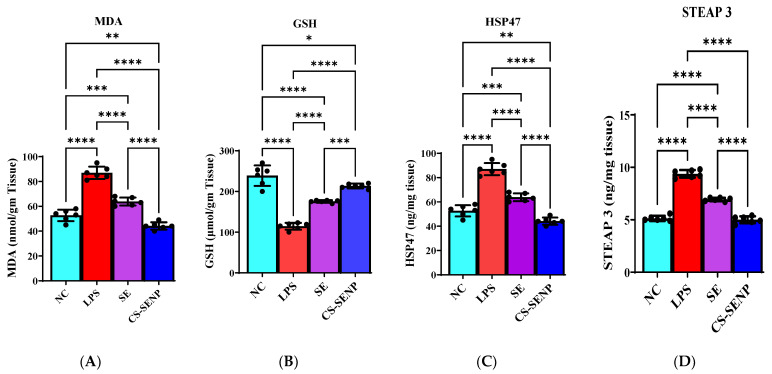
Effect of SE, and CS-SENPs on MDA (**A**), GSH (**B**), HSP-47 (**C**), and STEAP-3 (**D**) in LPS-exposed rats. Data are presented as mean ± SD of 5–6 pooled replicates (n = 10 rats per group). Pooling was performed before biochemical analysis. *p* < 0.05 was considered statistically significant. *: *p* < 0.05; **: *p* < 0.01; ***: *p* < 0.001; ****: *p* < 0.0001. LPSs: lipopolysaccharides, SE: Selenium, CS-SENPs: Chitosan-Coated Selenium Nanoparticles.

LPS severely induced oxidative stress in the liver, shown by a significant 2.2-fold increase in MDA levels and a 52% reduction in GSH levels compared to control rats (Figure 4A,B). SE and CS-SENP treatment reversed the change in oxidative stress biomarkers. SE and CS-SENPs reduced MDA levels by 30.27% and 61.7%, respectively, compared to the LPS group (Figure 4A). Conversely, SE and CS-SENPs increased GSH levels by 1.53-fold and 1.86-fold, respectively, compared to the LPS group (Figure 4B).

LPS significantly increased hepatic HSP-47 levels by 1.65-fold, while treatment using SE and CS-SENPs reduced hepatic HSP-47 levels by 26.6% and 49.3%, respectively (Figure 4C). Conversely, LPS significantly elevated STEAP-3 levels compared to the normal control group (1.8-fold increase). SE and CS-SENP treatment reversed the STEAP-3 increase, reducing its levels by 25.8% and 46.23%, respectively, compared to the LPS group (Figure 4D).

### 3.4. Effect of SE and CS-SENP Treatment on HO-1, TLR-4, STAT-3, TRAF-6, and IL-17A Levels

**Figure 5:**
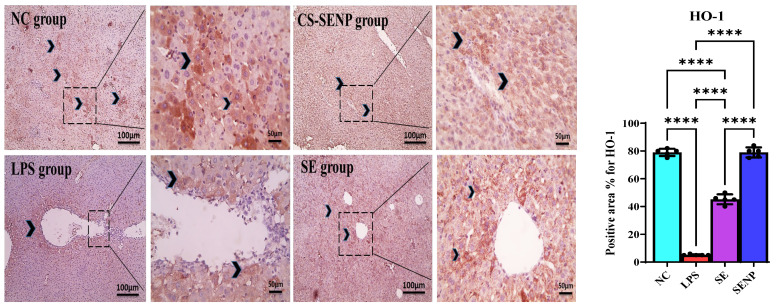
The immuno-stained liver sections against HO-1. Shows a marked positive HO-1 brown reaction in hepatocytes (black arrowheads) in the control group and the group that received CS-SENPs. Liver sections from the LPS group showed weak positive brown reaction against Ho-1 in hepatocytes (black arrowheads). Liver sections from the SE group showing increased positive brown reaction in hepatocytes against Ho-1 (black arrowheads). IHC counterstained with Mayer’s hematoxylin. Low magnification ×: 100 bar 100 and high magnification ×: 400 bar 50. *p* < 0.05 was considered statistically significant. ****: *p* < 0.0001. LPSs: lipopolysaccharides, SE: Selenium, CS-SENPs: Chitosan-Coated Selenium nanoparticles.

**Figure 6:**
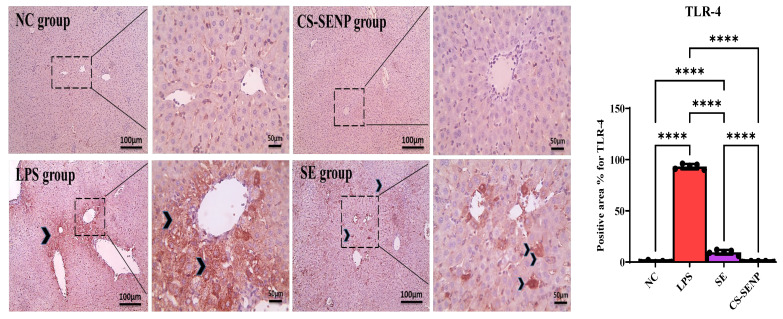
The immuno-stained liver sections against TLR-4. The figure shows a negative reaction in hepatocytes in the control group and the group that received CS-SENPs. Liver sections from the LPS group exhibited a strong brown positive immunoreaction for TLR-4 within hepatocytes (black arrowheads). In contrast, liver sections from the SE-treated group showed a markedly reduced TLR-4–positive brown signal in hepatocytes (black arrowheads). Sections were counterstained with Mayer’s hematoxylin. Low-magnification images: ×100 (scale bar = 100 µm); high-magnification images: ×400 (scale bar = 50 µm). Statistical significance was set at *p* < 0.05. **** *p* < 0.0001. LPSs: lipopolysaccharides; SE: selenium; CS-SENPs: chitosan-coated selenium nanoparticles.

Hepatocytes in the NC group and the CS-SENP group showed a noticeable positive brown reaction on immunohistochemical HO-1-stained slides. Hepatocytes in liver sections from the LPS group have a weakly positive brown response against HO-1. Hepatocytes in liver sections from the SE group had a greater positive brown reaction against HO-1 (Figure 5). Additionally, hepatocytes in the NC group and the group that received CS-SENPs exhibited a negative response on immunohistochemistry TLR-4-stained slides. Hepatocytes in liver sections from the LPS group had a robust positive brown reaction against TLR-4. Hepatocytes in liver sections from the SE group exhibited a significantly reduced positive brown reaction to TLR-4 (Figure 6).

In comparison to NC rats, LPS significantly reduced the HO-1 level by 93.4% and raised the TLR-4 protein level by 155.3 times. In comparison to the LPS group, SE and CS-SENPs raised HO-1 levels by 8.7 and 15.9 times, respectively. In contrast to the LPS group, SE and CS-SENPs decreased TLR-4 levels by 89.3% and 99.35%, respectively (Figure 5 and Figure 6).

**Figure 7:**
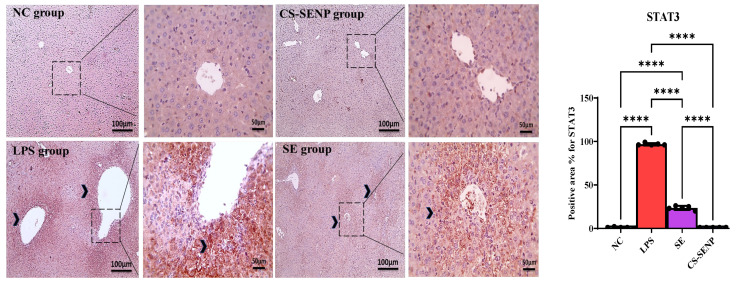
The immuno-stained liver sections against STAT-3. Shows a negative hepatocyte reaction to STAT-3 in the control group, while the LPS group shows a strong positive brown reaction in hepatocytes against STAT-3 (black arrowheads). Liver sections from the SE and CS-SENP group show a markedly decreased positive brown reaction in hepatocytes against STAT-3 (black arrowheads). IHC counterstained with Mayer’s hematoxylin. Low magnification ×: 100 bar 100 and high magnification ×: 400 bar 50. *p* < 0.05 was considered statistically significant. ****: *p* < 0.0001. LPSs: lipopolysaccharides, SE: Selenium, CS-SENPs: Chitosan-Coated Selenium nanoparticles.

**Figure 8:**
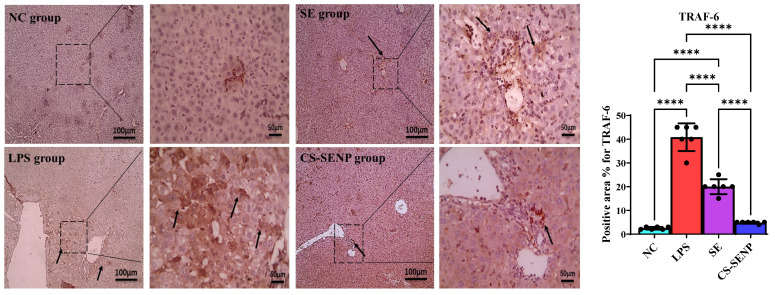
The immuno-stained liver sections against TRAF-6. Microscopic pictures of immuno-stained hepatic sections against TRAF-6 showing weak positive brown expression (arrow) in control N group, markedly increased positive brown expression in many hepatocytes associated with areas of portal fibrosis & inflammation (arrows) in LPS group, decreased positive brown expression in some hepatocytes (arrows) in treated group with SE, mild positive brown expression (arrows) in treated group with CS-SENPs. IHC counterstained with Mayer’s hematoxylin. Low magnification ×: 100 bar 100 and high magnification ×: 400 bar 50. *p* < 0.05 was considered statistically significant. ****: *p* < 0.0001. LPSs: lipopolysaccharides, SE: Selenium, CS-SENPs: Chitosan-coated Selenium nanoparticles.

Immunohistochemical STAT-3-stained slides show a negative reaction to STAT-3 in the control group, while the LPS group shows a strong positive brown reaction in hepatocytes against STAT-3. Liver sections from the SE and CS-SENP groups show a markedly decreased positive brown reaction in hepatocytes against STAT-3 (Figure 7). Besides, microscopic pictures of immunostained hepatic sections against TRAF-6 showed weak positive brown expression in the NC group, markedly increased positive brown expression in many hepatocytes associated with areas of portal fibrosis & inflammation in the LPS group, decreased positive brown expression in some hepatocytes in the treated group with SE, and mild positive brown expression in the treated group with CS-SENPs (Figure 8).

As shown in Figure 7 and Figure 8, the increased generation of inflammatory mediators was linked to LPS. In comparison to control rats, the LPS group had a significant increase in STAT-3 and TRAF-6 levels (↑ 96.8-fold and 16.32-fold, respectively). This was reversed in the SE-treated group (↓ 75.6% and 51%, respectively) and CS-SENP-treated group (↓ 99% and 88.2%, respectively) when compared to the LPS rats’ group.

**Figure 9:**
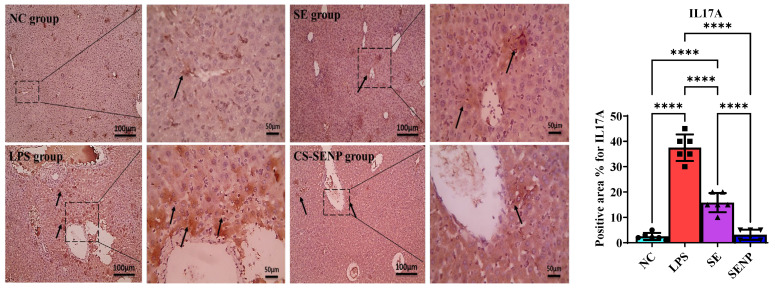
The immuno-stained liver sections against IL-17A. Microscopic pictures of immuno-stained hepatic sections against IL17A showing weak positive brown expression (arrow) in the control N group, markedly increased positive brown expression in many hepatocytes (arrows) associated with areas of portal fibrosis and inflammation in the LPS group. decreased positive brown expression in some hepatocytes (arrows) in the treated group with SE, and mild positive brown expression in a few hepatocytes (arrows) in the treated group with CS-SENPs. The IHC sections were counterstained with Mayer’s hematoxylin. Images were captured at low magnification (×100; scale bar = 100 µm) and high magnification (×400; scale bar = 50 µm). Statistical significance was defined as *p* < 0.05. ****: *p* < 0.0001. Abbreviations: LPSs, lipopolysaccharides; SE, selenium; CS-SENPs, chitosan-coated selenium nanoparticles.

Microscopic pictures of immunostained hepatic sections against IL-17A showing weak positive brown expression in the NC group and markedly increased positive brown expression in many hepatocytes associated with areas of portal fibrosis and inflammation in the LPS group. Decreased positive brown expression was observed in some hepatocytes in the treated group with SE, and mild positive brown expression was detected in a few hepatocytes in the treated group with CS-SENPs. LPS significantly increased hepatic IL-17A levels by 15-fold, while treatment using SE and CS-SENPs reduced hepatic IL-17A levels by 57.8% and 91.5%, respectively (Figure 9).

### 3.5. Effect of SE and CS-SENP Treatment on HSP-90

**Figure 10:**
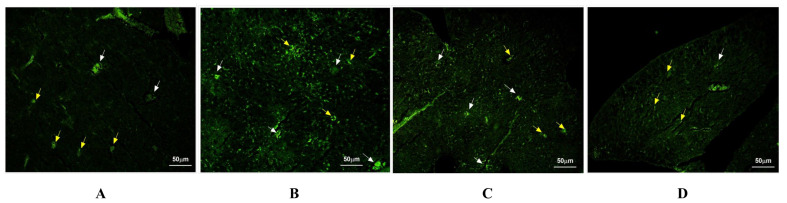
The immuno-stained liver sections against HSP90. (**A**) The normal control group showed a weak expression of HSP-90 with mild fluorescence intensity (+) compared to the LPS-treated tissue. The HSP-90 protein showed membranous and cytoplasmic expression (yellow arrowhead) in hepatocytes (yellow arrowhead) and in Kupffer cells (resident macrophages) as pointed by the white arrow. (**B**) The LPS-treated tissue showed a significantly high expression of HSP-90 with high fluorescence intensity (+3) compared to the healthy group. The HSP-90 protein showed membranous and cytoplasmic expression in hepatocytes (yellow arrowhead) and Kupffer cells (resident macrophages) as pointed by the white arrow. (**C**) SE-treated tissue showed a significant decrease in the expression of HSP-90 with moderate fluorescence intensity (+2) compared to the LPS-treated group. The HSP-90 protein showed membranous and cytoplasmic expression in hepatocytes (yellow arrowhead) and in Kupffer cells (resident macrophages), as pointed by the white arrow. (**D**) CS-SENP-treated tissue showed a marked decrease in the expression of HSP-90 with mild to moderate fluorescence intensity (+1) compared to the LPS-treated group. The HSP-90 protein showed membranous and cytoplasmic expression in hepatocytes (yellow arrowhead) and in Kupffer cells (resident macrophages) as pointed by the white arrow. The magnification is 40×.

The LPS-treated tissue showed a significantly high expression of HSP-90 with high fluorescence intensity compared to the healthy group (Figure 10). SE-treated tissue showed a significant decrease in the expression of HSP-90 with moderate fluorescence intensity compared to the LPS-treated group. CS-SENP-treated tissue showed a marked decrease in the expression of HSP-90 with mild to moderate fluorescence intensity compared to the LPS-treated group. The HSP90 protein showed membranous and cytoplasmic expression in hepatocytes and Kupffer cells (resident macrophages). LPS significantly increased hepatic HSP-90 levels by 12.11-fold, while treatment using SE and CS-SENPs reduced hepatic HSP-90 levels by 31.7% and 65.66%, respectively (Figure 10).

## 4. Discussion

The physicochemical evaluation of the synthesized CS-SENPs confirmed the successful formation and colloidal stability of the prepared CS-SENPs. The hydrodynamic diameter of the obtained CS-SENPs (108.54 ± 2.24 nm) with their low PDI (0.125 ± 0.04) suggests a narrowly distributed, monodisperse nanoparticulate system, which is critical for pharmaceutical applications, as nanoparticles less than 200 nm exhibit optimal tissue penetration and permeation characteristics through biological membranes [38,39]. Since values above ±30 mV are linked to resistance to aggregation, the extremely positive zeta potential of +64 ± 6.287 mV implies significant electrostatic repulsion between particles, which is widely acknowledged as a key factor of long-term colloidal stability. The amino groups (−NH_3_^+^) found in CS have been identified as the cause of similar positive surface charge magnitudes reported for CS-SENPs [40,41]. Through electrostatic interactions with the negatively charged cellular membrane, this noticeable positive charge provides additional utility for possible cellular uptake [42,43]. Further confirming the establishment of a core–shell architecture in which CS creates a stabilizing interfacial layer, the TEM micrograph revealed distinct, spherical particles with smooth outlines and a visible electron-lucent corona encircling the SENP core. These kinds of core–shell morphologies have been frequently reported for CS-SENPs and are linked to better biological performance and colloidal behavior when compared to bare SENPs [44,45].

CS-SENPs had better therapeutic efficacy than SE. This enhanced efficacy is mechanistically attributable to multiple complementary advantages of the CS coating: (1) the positive surface charge of CS enables electrostatic interaction with negatively charged hepatocyte membranes and preferential uptake by Kupffer cells, facilitating hepatic accumulation [24,25]; (2) the nanoparticle architecture provides sustained SE release, reducing peak toxicity while prolonging selenoprotein (GPx, TrxR) synthesis.

SE in CS-SENPs is not chemically bonded to CS in a simple cleavable linkage, it is physically adsorbed within and on the CS polymeric corona surrounding the SENP core. Release occurs through several overlapping mechanisms: SE ions/atoms diffuse outward through the CS matrix as a concentration gradient is established between the nanoparticle and the surrounding medium. It has recently been demonstrated that CS-SENPs shield cells against SE-induced DNA damage [46]. Additionally, shell nanocapsules that target cancer cells and improve medication release over extended periods of time under acidifying stimuli (pH 5.3) have been created by decorating SENPs with folate-CS [47]. Furthermore, endocytosis pathways allowed cells to absorb SENPs, which had outstanding biological functions [48]. The release kinetics of SE from CS-SENPs have been described in a number of investigations as a regulated biphasic process. The system shows an initial burst release in physiological buffers (PBS, pH 7.4), followed by a protracted sustained delivery phase lasting up to 48 h [49]. The quick diffusion of surface-associated SE and the slow swelling and structural deterioration of the CS matrix are the two mechanisms that control this profile. This controlled release is essential for preserving SE within the therapeutic window, optimizing bioavailability, and effectively reducing the risks of systemic toxicity brought on by the quick “spikes” in free SE forms [49,50].

Complementary evidence for effective surface functionalization and interaction between CS and SENPs is provided by the FTIR profile of CS-SENPs. The creation of intimate interfacial contact between the CS and the SENP core is strongly supported by the broad and intense absorption at 3426 cm^−1^, which is caused by the overlapping stretching vibration of the absorption peak of the OH group of SENPs and the N-H of CS [51,52]. Strong surface immobilization and improved structural organization at the polymer-nanoparticle interface are made possible by this spectrum manifestation, which stands for improved coordination chemistry and complexation events between the amino group of CS and the OH group of SENPs. Through the many hydroxyl and amino functionalities of CS, the polymeric coating significantly improves the hydrophilic nature of the nanoparticle surface, encouraging remarkable dispersibility in aqueous media and averting hydrophobic aggregation phenomena frequently seen in uncoated SENP systems [53]. The presence and position of the CS macromolecular scaffold on the SENP periphery are supported spectroscopically by the C-H stretching vibrations found at 3024, 2926, and 2607 cm^−1^ [28]. These unique vibrational modes produce a spectroscopic fingerprint that confirms the presence of CS in the nanoparticle coating architecture and shows an ordered conformational arrangement of the polymer chains upon surface adsorption. The amide bands at 1648.84 and 1415.49 cm^−1^ represent the acetyl-substituted glucosamine moieties in the CS fundamental structure. This suggests that CS’s acetamide functions maintain their structural integrity following the production of nanoparticles, which is essential for maintaining its capacity to form hydrogen bonds and interact with biological media [54,55,56]. This suggests that CS’s acetamide functions maintain their structural integrity following the production of nanoparticles, which is essential for maintaining its capacity to form hydrogen bonds and interact with biological media [40,41,57]. According to the results, CS creates a monodisperse and extremely stable nanocarrier platform by forming a positively charged corona surrounding SENPs through SE-O-C interfacial interactions.

LPS, a powerful endotoxin that causes severe oxidative and inflammatory reactions, is frequently used to imitate ALI. The liver is especially susceptible to LPS-induced damage, which shows oxidative stress, cytokine storm, ferroptosis, and deregulation of stress response proteins. The liver is a key organ in detoxification and immunological surveillance [58]. The dose of CS-SENPs was selected based on prior toxicological and pharmacological studies. Specifically, SENPs have been shown to be significantly less toxic than inorganic SE, with an LD50 of 198.1 mg/kg and a proven safety profile at doses up to 10 mg/kg [35]. Furthermore, SENPs at similar concentrations have demonstrated potent immunomodulatory and antioxidant activities in various disease models [34]. Therefore, the current dose was chosen to ensure optimal therapeutic efficacy while maintaining a wide margin of safety. The results showed that CS-SENPs have better therapeutic efficacy than SE. This is probably because CS’s advantageous biodistribution and SENPs’ antioxidant capacity work together to improve bioavailability and cellular uptake.

Oxidative stress was one of the primary pathogenic features of liver injury, as shown by elevated MDA and lowered GSH. The increased ROS-mediated lipid peroxidation of hepatic polyunsaturated fatty acids, which spreads membrane damage and hepatotoxicity, is reflected in the 2.2-fold increase in MDA under LPS. Although limited by poor dispersibility and quick clearance from hepatic sinusoids, partial GPx activation is the cause of SE’s mild 30.27% attenuation. By utilizing CS’s stability for improved bioavailability, targeted Kupffer cell delivery, and sustained selenoprotein synthesis, CS-SENPs’ 61.7% suppression, which restores levels close to NC, breaks down peroxide chains more thoroughly [59,60]. By incorporating selenocysteine into GPx, which recycles glutathione disulfide back into GSH, SE partially restores GSH to intermediate levels (1.53-fold compared to LPS). Through CS’s coating, which improves nanoparticle stability, sinusoidal retention, and prolonged GPx/TrxR expression, CS-SENPs surpass SE by raising GSH to levels close to NC. In order to achieve complete redox equilibrium, this formulation increases de novo GSH synthesis through upregulated glutamate-cysteine ligase while reducing the consumption of ROS [61]. As a result, after being exposed to LPS, the cytoprotective enzyme HO-1, which has anti-inflammatory and antioxidant properties, was decreased. Notably, normalization of HO-1 by SE and CS-SENPs indicates restoration of cellular homeostasis and decreased requirement for stress-triggered protective systems; HO-1 overexpression in acute stress may represent a compensatory reaction [62,63,64]. These results demonstrated that CS-SENPs were a dual regulator of redox equilibrium that simultaneously strengthens endogenous antioxidant defenses and attenuates pro-oxidant drives.

Additional proof of this antioxidant effect was obtained through modification of STEAP-3. The documented rise in STEAP-3 following LPS treatment indicates that STEAP-3 is an important regulator of oxidative stress and iron metabolism. By acting as a ferrireductase, STEAP-3 overexpression directly worsens oxidative imbalance and hepatocellular damage under inflammatory circumstances [8]. SE treatment significantly decreased STEAP-3 by regulating redox equilibrium, boosting antioxidant enzymes, and lowering oxidative stress signaling [65,66]. However, CS-SENPs were more effective than SE. This enhanced effect is due to CS-SENPs’ nanoscale size, which increases SE bioavailability and intracellular transport. Additionally, CS adds anti-inflammatory and biocompatible properties to strengthen the antifibrotic activity [67]. At the molecular level, CS-SENPs downregulated STEAP-3 and markedly elevated HO-1. These results demonstrate that CS-SENPs are a dual-action medication that targets both redox imbalance and iron dysregulation, necessitating further mechanistic research and dose optimization in models of chronic inflammatory disorders.

Another characteristic of LPS-induced hepatic insult is inflammation, which is mainly caused by TLR-4 activation and subsequent signaling cascades. Pro-inflammatory cytokines and chemokines are released when endotoxin engages TLR-4, activating the NF-κB and MAPK pathways. TLR-4 expression was considerably decreased by SE and CS-SENPs, suggesting a reduction in the original inflammatory trigger [68,69]. Compared to SE, CS-SENPs produced a more profound suppression, demonstrating the improved effectiveness of the nanoparticle formulation. Reduced levels of TRAF-6 and IL-17, important mediators in the IL-17 signaling axis, coincided with this inhibition. Th17 cells are the primary source of IL-17, which increases inflammation by triggering chemokines and cytokines via TRAF-6-dependent activation of the NF-κB and MAPK pathways [70,71]. As an adapter and E3 ubiquitin ligase, TRAF-6 promotes the spread of IL-17 signals, which leads to the recruitment of immune cells and tissue damage. TRAF-6 and IL-17 were downregulated in rats given SE and CS-SENPs, demonstrating their ability to disrupt this pro-inflammatory loop and lessen immune-mediated hepatotoxicity [72,73]. Another crucial inflammatory mediator, STAT-3, was significantly elevated in livers treated with LPS. The transcription factor STAT-3, which encourages fibrogenesis, inflammation, and cell survival, is stimulated by growth factors and cytokines. Its decrease by CS-SENPs and SE implies more widespread regulation of cytokine signaling networks, which further reduces liver damage. Since IL-17 can activate STAT-3 through intermediary pathways, increasing inflammatory and fibrotic responses, this action may intersect with the IL-17/TRAF-6 axis. Therefore, it seems that SE has a coordinated inhibitory effect on several inflammatory nodes [74,75].

SE affected the production of stress-related proteins, particularly HSP-90 and HSP-47, in addition to its impacts on cytokine signaling. Both indicators were significantly elevated by the LPS challenge, indicating the activation of stress-protein networks and collagen chaperone machinery that support inflammatory signaling. These alterations were lessened by SE supplementation, and the antioxidant and anti-inflammatory properties of SE and CS-SENPs decreased HSP-90 expression. Since HSP-90 is a molecular chaperone that stabilizes several client proteins in the NFκκB and MAPK pathways, pro-inflammatory mediators like IL-17, TRAF-6, and TLR-4, which rely on HSP-90 for signaling stability, are probably inhibited upstream by SE. This mechanistic connection is supported by the observed decrease in HSP-90 after SE and CS-SENP therapy, indicating that SE indirectly downregulates HSP-90 by reducing oxidative and inflammatory stress [43].

LPS-induced hepatic damage is modulated in a variety of ways by SE and CS-SENPs. Their protective effects include reduction in MDA, restoration of GSH, and induction of HO-1; regulation of iron metabolism through suppression of STEAP-3 to limit ferroptosis-like oxidative cascades; inhibition of inflammatory signaling through downregulation of TLR-4, IL-17, TRAF-6, and STAT-3; modulation of stress proteins through suppression of HSP-90 and HSP-47, thereby reducing chaperone-driven inflammation, fibrosis, and vascular congestion.

Moreover, CS-SENPs’ higher efficacy over inorganic SE is due to their nanoscale architecture, which enhances bioavailability, encourages cellular absorption, and keeps selenoproteins like TrxR and GPx activated. These features enable CS-SENPs to concurrently lower oxidative stress, inflammatory signaling, and fibrogenic remodeling, three interrelated mechanisms crucial to endotoxemia-induced liver damage. CS-SENPs are a potential therapy option for sepsis-associated liver damage, a condition where conventional antioxidants often fail, due to their restricted tissue accumulation. By controlling immunological and metabolic reactions, CS-SENPs guard against acute hepatocellular damage and stop the onset of chronic liver diseases including cirrhosis and fibrosis.

### Study Limitations

Several limitations of the present study should be acknowledged. First, pharmacokinetic and biodistribution studies were not performed, which limits mechanistic conclusions regarding the precise hepatic accumulation, peak concentration, and clearance kinetics of CS-SENPs in vivo; future studies should incorporate such analyses using radiolabeling or fluorescence tracking. Second, a free selenium + chitosan control group was not included in the original experimental design, which precludes complete isolation of the nanoparticle-specific effect from potential independent contributions of chitosan; this control should be included in future investigations. Third, the mechanistic pathway analysis was conducted primarily at the protein level using ELISA, IHC, and IF; future studies should validate findings using Western blot and qPCR for mRNA-level quantification to further consolidate the mechanistic conclusions. Fourth, the study was conducted exclusively in male rats, which may limit generalizability to female subjects or to humans. Fifth, in vitro cell-based mechanistic studies were not included and would be valuable for delineating intracellular signaling in greater detail.

## 5. Conclusions

The study highlights the potent hepatoprotective action of SE, particularly in its nanoparticle form. CS-SENPs performed better than inorganic SE in decreasing LPS-induced acute liver damage. CS-SENPs mechanistically restored redox balance by increasing GSH, decreasing MDA, and activating HO-1. Their simultaneous reduction in STEAP-3 decreased iron-driven oxidative cascades and ferroptosis. Inflammatory mediators such as TLR-4, IL-17, TRAF-6, and STAT-3 were significantly downregulated. Chaperone-induced inflammation and fibrogenesis were lessened by the reduction in stress proteins HSP-90 and HSP-47. Histological analysis confirmed nearly normal hepatic architecture in rats given CS-SENPs. The multitargeted activity of CS-SENPs spanning oxidative, inflammatory, and fibrotic pathways is highlighted by these combined effects. These characteristics make CS-SENPs a promising treatment option for liver damage brought on by sepsis.

## Data Availability

The original contributions presented in this study are included in the article. Further inquiries can be directed to the corresponding authors.

## Abbreviations

The following abbreviations are used in this manuscript:

SE	Selenium
CS-SENPs	Chitosan-coated selenium nanoparticles
LPS	lipopolysaccharide
PDI	Polydispersity index
TEM	Transmission electron microscope
FTIR	Fourier transform infrared spectroscopy
ALI	Acute liver injury
HSP	Heat shock protein
TrxR	Thioredoxin reductase
GPx	Glutathione peroxidase
MDA	Malondialdehyde
IL-17	Interleukin-17
